# A modified two-step technique for the retrieval of a chemoport catheter fragment with inaccesible ends

**DOI:** 10.1186/s42155-022-00342-x

**Published:** 2022-12-05

**Authors:** Naushad K Sokwalla, Ravjit Sagoo, Alel Moussa, Manel Haj Mansour

**Affiliations:** 1 Department of Radiology, Aga Khan University Hospital Nairobi, Nairobi, Kenya; 2 Department of Oncology, Aga Khan University Hospital Nairobi, Nairobi, Kenya

## Abstract

**Background:**

Migration of central venous catheters is a rare but serious complication. The endovascular approach has been widely used for the retrieval of such fragment, with the two-step technique used for removal of catheter fragments with inaccessible ends. In this case report, we describe a modification of this technique that was used after first attempting the two-step technique unsuccessfully.

**Case presentation:**

A 42-year-old female with breast cancer had a chemoport inserted for chemotherapy. After 6 cycles of chemotherapy the port could not be flushed and a chest radiograph demonstrated a migrated catheter fragment. CT scan demonstrated that one end of the fragment was in the liver in the middle hepatic vein and the other in the right atrial appendage. A modified 2 step technique, using a pigtail catheter, hydrophilic wire and snare was used to remove this fragment.

**Conclusion:**

In this case report we highlight a new modification of the 2-step technique that can be employed when the conventional 2 step technique does not work.

## Background

Migration of central venous catheter fragments is a rare but severe complication. The endovascular approach is widely used for retrieval of foreign bodies, commonly using the snare loop catheter. When no free ends are available to snare, a two-step technique has been described. Firstly, a pigtail catheter is used to hook around the displaced catheter fragment and pulled to free an end. Subsequently a snare catheter is used to capture the free end and retrieve the catheter fragment. Here we describe a modification of this technique, whereby the catheter fragment was firmly wedged with one end in the distal middle hepatic vein and the other end in the right atrial appendage and a pigtail catheter could not be used to create a free end.

## Case presentation

**Fig. 1:**
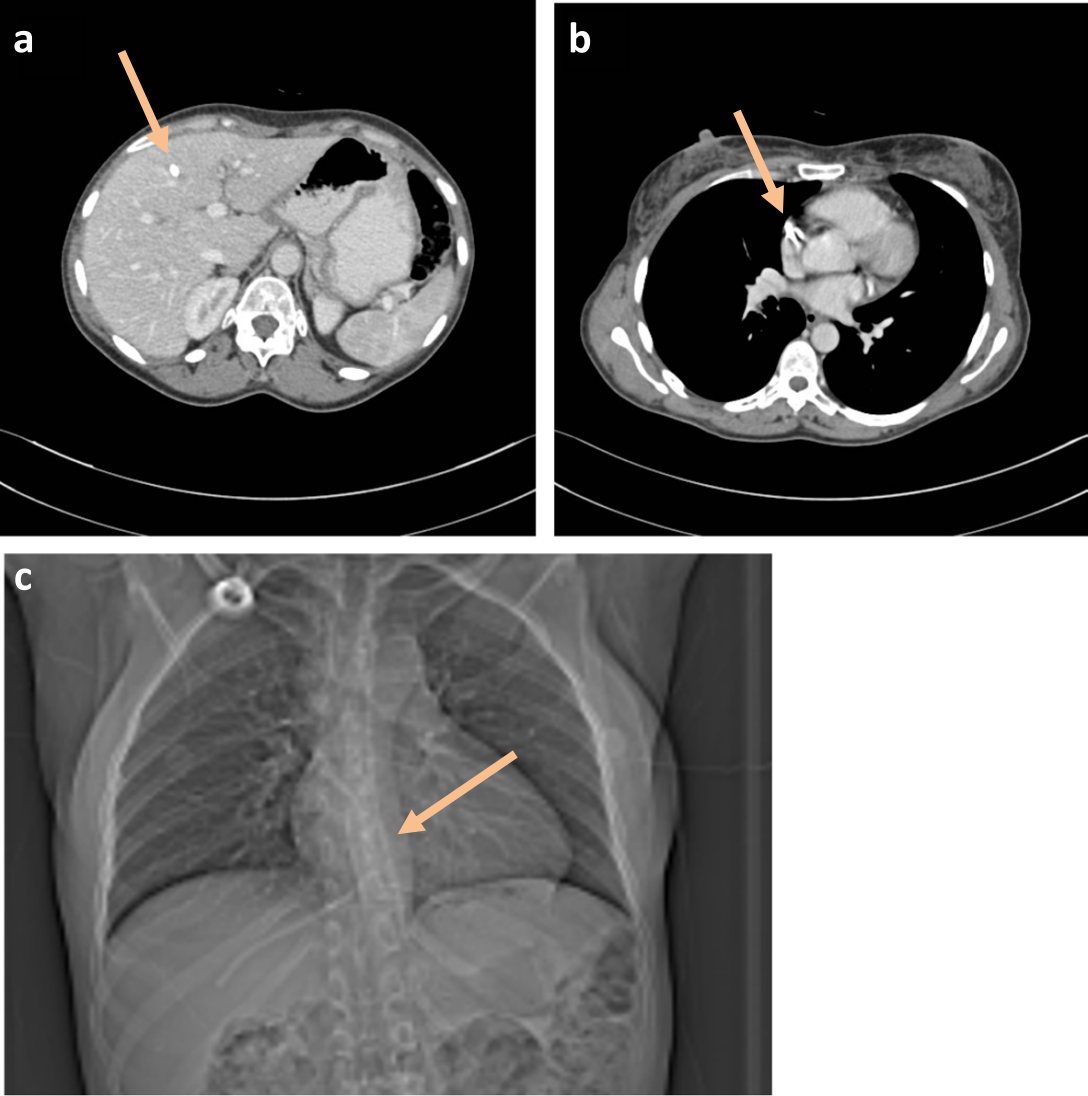
Contrast enhanced axial images of the chest and abdomen demonstrating the distal tip of the catheter fragment in the middle hepatic vein (**a**, orange arrow) and proximal tip in the right atrial appendage (**b**, orange arrow). Scanogram of the CT chest and abdomen demonstrating the catheter fragment (**c**, orange arrow)

A 42-year-old female with breast cancer had a chemo-port inserted in June 2022. This was used for 6 cycles of chemotherapy, after which the port could not be flushed. A chest radiograph revealed that the chemo-port catheter tubing had displaced with one end in the hepatic vein and the other end in the right side of the heart. She presented to our institution for removal of the catheter fragment and was otherwise asymptomatic. A contrast enhanced CT chest and abdomen was carried out demonstrating the displaced catheter fragment with one end in the middle hepatic vein (Fig. 1a) and the other end in the right atrial appendage (Fig. 1b).

**Fig. 2:**
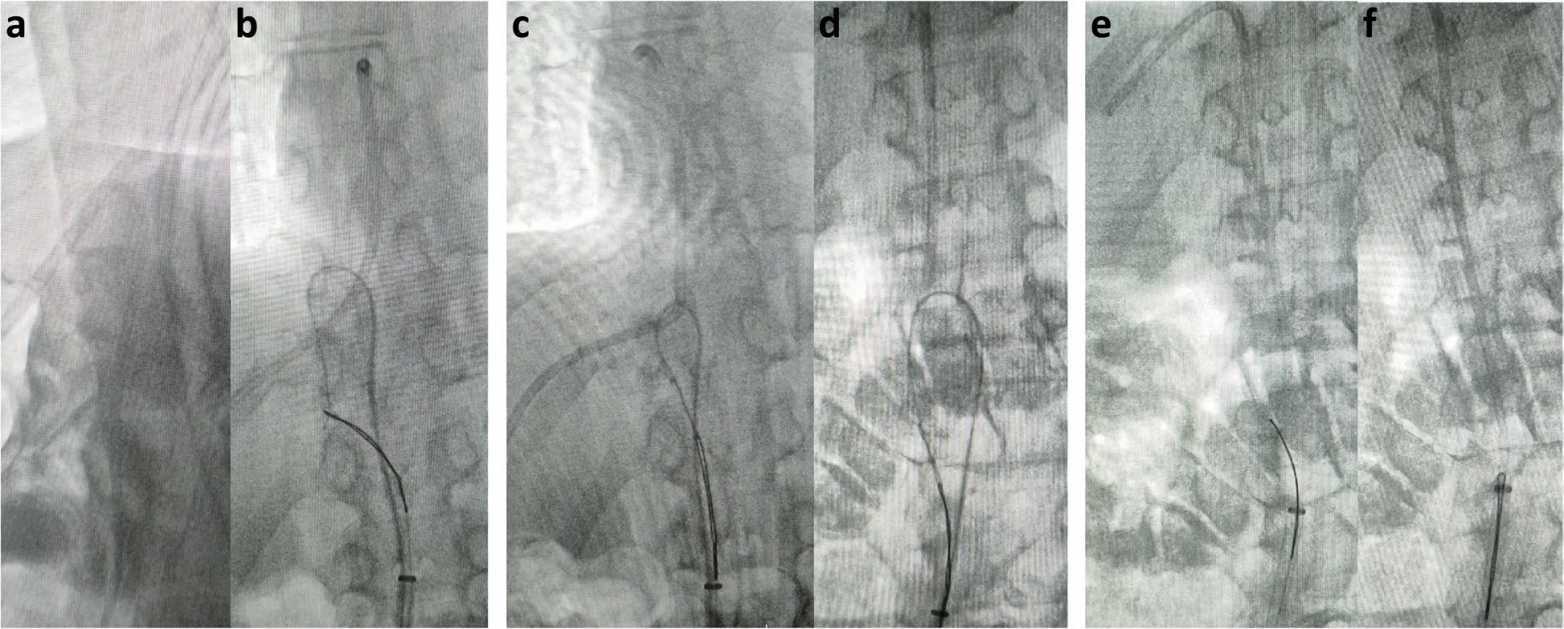
Lateral fluoroscopy image demonstrating placement of the pigtail catheter around the catheter fragment (**a**). AP fluoroscopic image demonstrating the snaring of the hydrophilic glidewire (**b**), followed by pulling of the pigtail catheter and snare catheter (**c**), to free the cardiac end of the catheter fragment (**d**). This was then snared and removed (**e**, **f**)

Percutaneous retrieval was performed as follows: under local anesthesia and ultrasound guidance, the right common femoral vein was punctured and a 12 Fr 28 cm sheath (Sentrant introducer sheath, Medtronic, Minneapolis, MN, USA) inserted into the inferior vena cava. A 20 mm Gooseneck snare (Amplatz Gooseneck snare, Medtronic, Minneapolis, MN, USA) was inserted and a 5 Fr angled pigtail catheter (Alvision, Alvimedica, Istanbul, Turkey) inserted through this, using the previously described “pigtail through snare” technique. Attempts were made to free an end of the displaced catheter fragment, however, the pigtail catheter kept unfolding over the displaced catheter fragment. The pigtail catheter was then removed and placed side by side with the snare catheter. With the pigtail catheter in place over the displaced catheter fragment (Fig. 2a), a hydrophilic glidewire (Blackeel hydrophilic glidewire, APT Medical, P. R. China) was advanced through the pigtail catheter and the free end of the wire snared (Fig. 2b). Since the wire could not be very firmly grasped and pulled by the snare because of its hydrophilic coating, it was held in place by the snare and the pigtail catheter and snare catheter pulled to create a free end of the displaced catheter fragment (Fig. 2c). The end of the displaced catheter fragment that was in the right atrium was freed and dropped into the inferior vena cava (Fig. 2d). The snare that was used to hold the wire was released and used to snare the free end of the displaced catheter fragment (Fig. 2e). The pigtail catheter was then removed from the sheath followed by the snare catheter with the catheter fragment (Fig. 2f). Haemostasis was achieved by manual compression.

## Discussion

Central venous catheter fragments have been reported to cause complications such as arrhythmias, perforation, clotting, infection and even death, and should be removed even if the patient is asymptomatic (Fisher and Ferreyro 1978).

The two-step technique using a pigtail catheter and a snare loop catheter in retrieving a dislodged catheter fragment with no accessible free ends was first described by Greenfield et al. in 1978 (Greenfield et al. 1978). The pigtail catheter is used to make at least one end free that can then be grasped by the snare catheter. Many interventional radiologists have reported on the usefulness of the two-step method, however, one problem of the two-step method is that sometimes, once freed, the free end would pass into the heart and once again become inaccessible before or during the snaring procedure (Bessoud et al. 2003; Rodrigues et al. 2007; Chuang et al. 2011; Pandey et al. 2018). A modified two-step “pigtail through snare” technique has recently been described to minimize the chances of this happening (Mori et al. 2021). Another modification of the two-step technique has also been recently reported, whereby a catheter fragment with inaccessible ends in the right atrium and ventricle was retrieved by crossing a wire across the fragment in the right ventricle and returning it to the right atrium where it was snared (Haga et al. 2020).

**Fig. 3:**
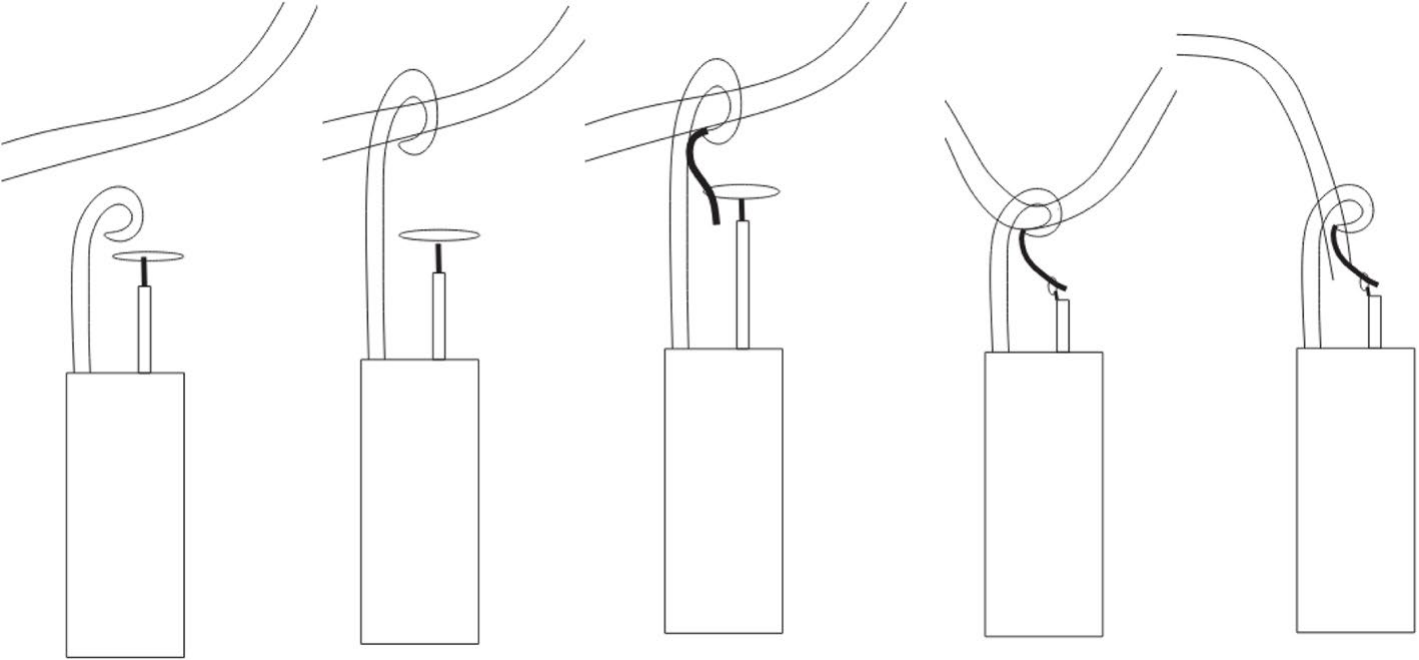
Schematic diagram demonstrating the modified two-step technique that was used in this case. The pigtail catheter and snare catheter were [laced side by side (**a**), followed by hooking the pigtail catheter around the catheter fragment (**b**). A hydrophilic glidewire was then inserted through the pigtail catheter and snared (**c**, **d**).The pigtail catheter and snare catheter were then pulled down to create a free end (**e**) which was then snared

In the present case we initially tried the modified two step “pigtail through snare” technique, however, the pigtail catheter kept unfolding over the displaced catheter fragment. We then resorted to modifying the original two-step technique by placing the pigtail catheter and snare catheter side to side (Fig. 3a), then hooking the pigtail catheter around the catheter fragment (Fig. 3b), followed by passing a Terumo hydrophilic glidewire through the pigtail and snaring it (Fig. 3c). The pigtail catheter and snare catheter were then pulled to free an end of the catheter fragment (Fig. 3d and e).

Whilst passing an adequate length of the hydrophilic glidewire through the pigtail catheter without unfolding it and snaring and holding a hydrophilic glidewire with enough traction without the wire slipping out can be technically challenging, this technique can be considered when difficulty is encountered in making an end of the displaced catheter fragment free with the use of a pigtail catheter alone. This technique can result in the freed end passing back into the heart and becoming inaccessible, as there is some time taken in disengaging the wire from the snare and then snaring the free end of the catheter fragment, and we would only advocate it for those cases where the pigtail catheter was not able to free an end of the catheter fragment.

## Conclusion

Retrieval of central venous catheter fragments should be carried out as they can be associated with serious complications. The two-step technique has been widely used successfully for retrieval of fragments that have no accessible free ends. In this case report, we describe a modification of this technique which can be used to free an end of the catheter fragment when a pigtail catheter alone is unable to achieve this.

## Data Availability

Not applicable.

## Abbreviations

CT: Computed tomography
MN: Minnesota
USA: United States of America
P. R. China: Peoples Republic of China
